# Supplementary material for: Future sea-level projections with a coupled atmosphere-ocean-ice-sheet model
Source: Nat Commun. 2023 Feb 14;14:636. doi: 10.1038/s41467-023-36051-9 (PMC9929224; doi:10.1038/s41467-023-36051-9)
Supplement: Supplementary file 1 — Supplementary Information [file 41467_2023_36051_MOESM1_ESM.pdf]

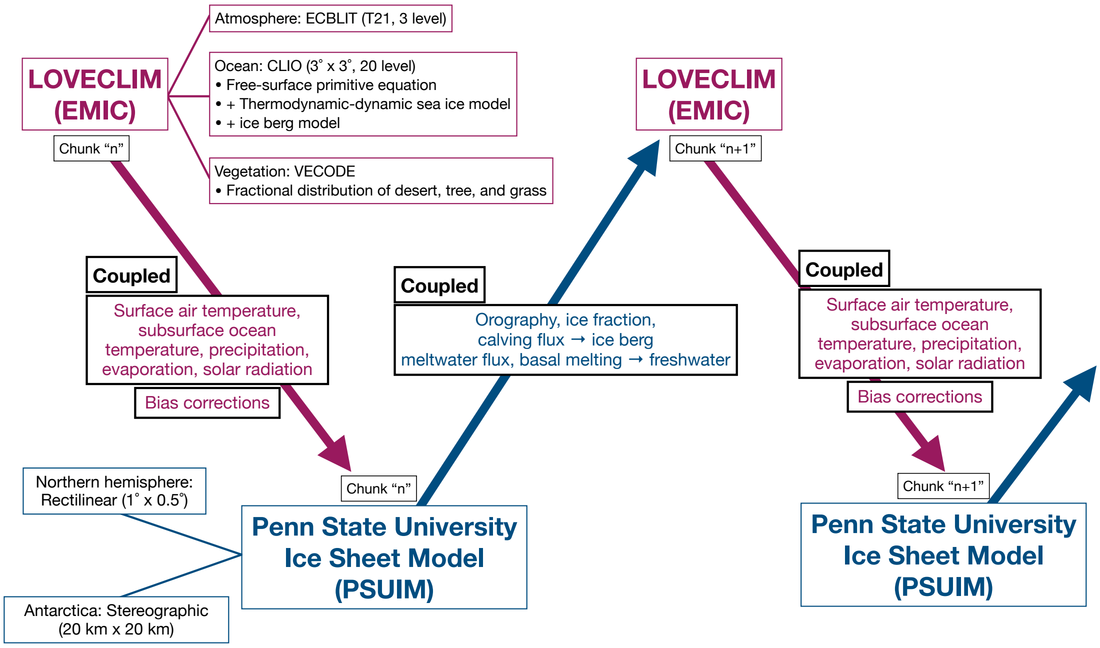

Supplementary figure S1 | Coupling algorithm. This schematic shows how the LOVECLIP is coupled and exchanges variables and boundary conditions between LOVECLIM and PSUIM.

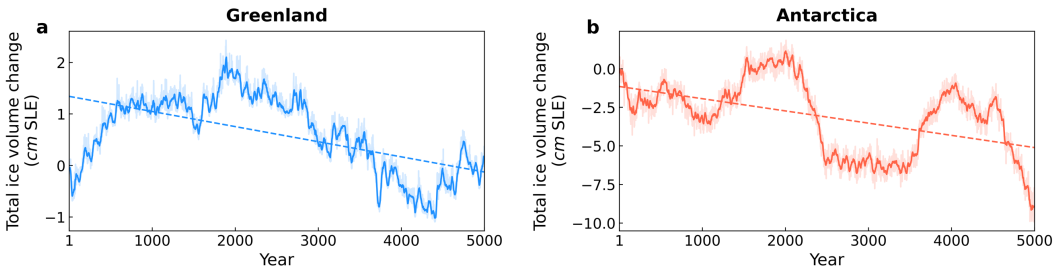

Supplementary figure S2 | Ice-sheet volume change in control experiment with pre-industrial CO_2 concentrations. (a) total ice-sheet volume change (relative to initial condition) over Greenland simulated by control experiment with constant, pre-industrial CO_2 concentrations; (b) same as (a), but for Antarctica. Shading indicates ensemble range, solid line the 19-year moving average of time series result and dashed line the linear regression by the Least Squares Method.

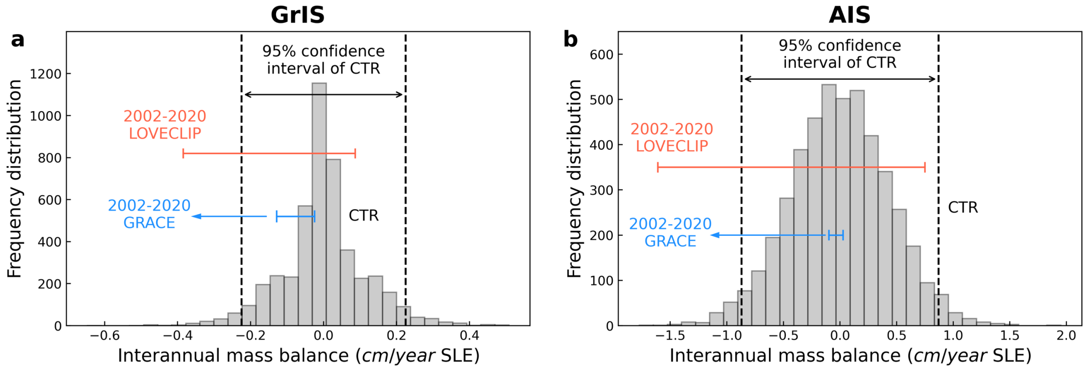

Supplementary figure S3 | Observed and simulated interannual mass balance of Greenland ice-sheet (GrIS) and Antarctic ice-sheet (AIS). (a) the histogram of Greenland interannual mass balance in the 5,000-year-long pre-industrial control run (CTR, gray histogram) with the 95% confidence interval range (black dashed line), and observed estimates for interannual change during 2002-2020 CE from the Gravity Recovery and Climate Experiment (GRACE) (blue line) and simulated by the forced LOVECLIP ensemble (red line); (b) same as (a), but for Antarctica. Consistent with the GRACE measurements, mass balance changes for LOVECLIM are calculated in this figure using only the grounded ice-sheet portion.

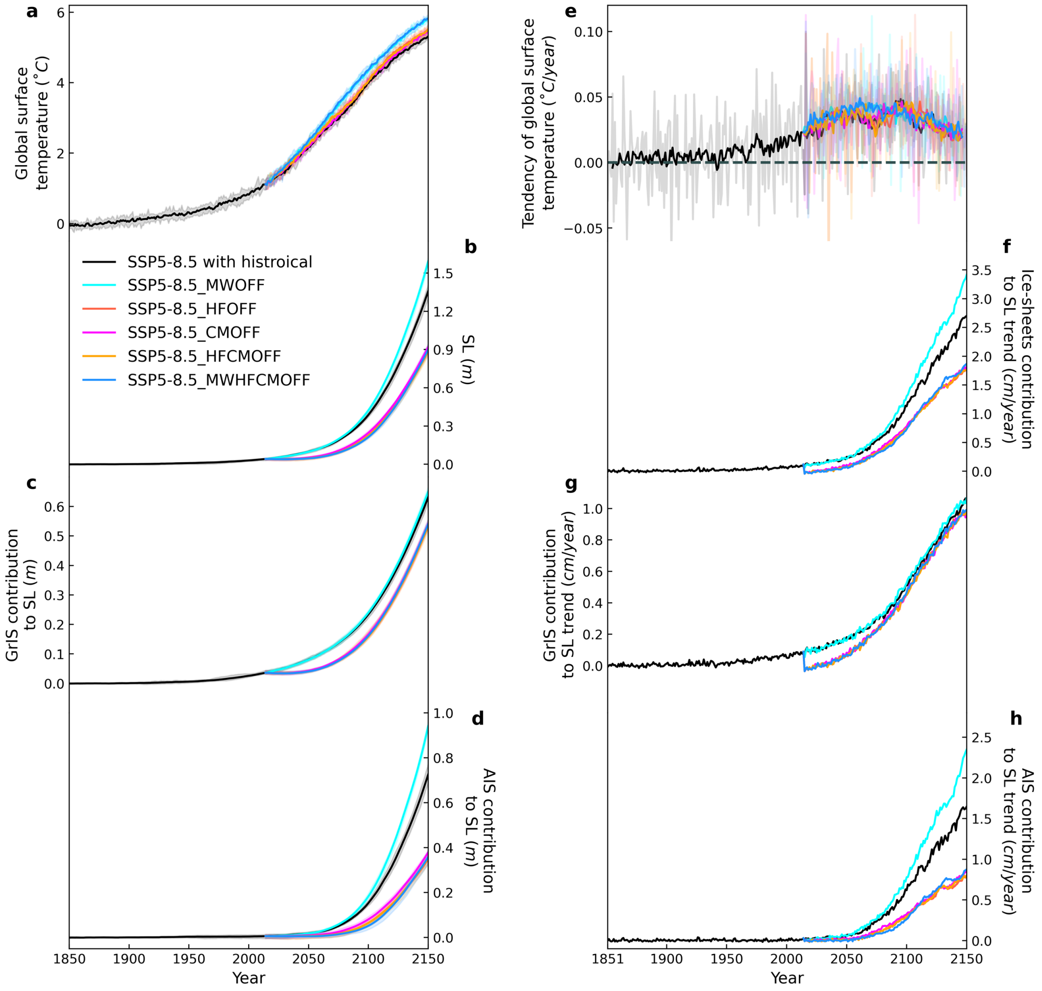

Supplementary figure S4 | Global surface temperature and sea-level (SL) projections, and their tendencies. (a-d) annual anomalies (relative to the 1850–1900 CE mean) of (a) the global surface temperature, (b) SL, and (c) SL contributions from the Greenland ice-sheet (GrIS) and (d) Antarctic ice-sheet (AIS). (e-h) are the respective time derivatives of (a-d) (change per year). Solid lines of (a-d) indicate the ensemble mean and shading the ensemble range. The solid line in (e) represents the 9-year moving average of the time derivative of global surface temperature, with the dashed line indicating 0 °C/year. Different colors represent the SSP5-8.5 with historical (black line; period 1850–2150 CE), and SSP5-8.5_MWOFF (cyan line), SSP5-8.5_HFOFF (red line), SSP5-8.5_CMOFF (pink line), SSP5-8.5_HFCMOFF (orange line) and SSP5-8.5_MWHFCMOFF (blue line) simulations during the period 2014–2150 CE. The sudden drops seen in (f and g) are due to the changes in parameters associated with hydrofracturing and ice-cliff failure.

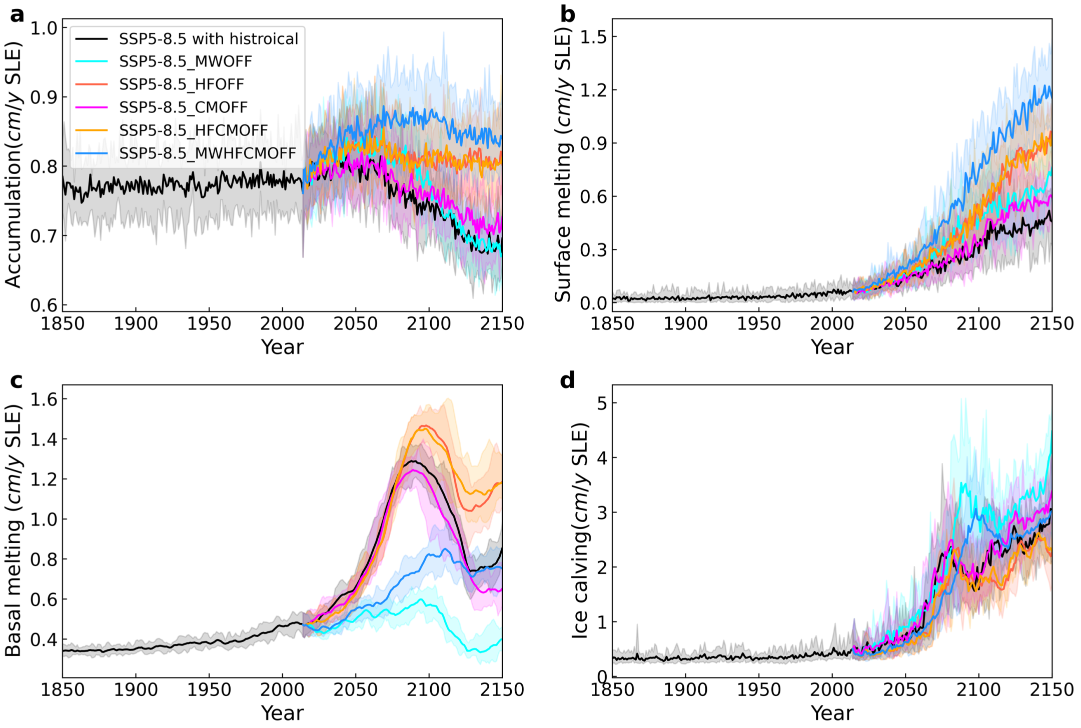

Supplementary figure S5 | Individual mass balance terms for Antarctic ice-sheet (AIS). (a-d) represent the individual AIS mass balance terms for (a) the accumulation, (b) surface melting, (c) basal melting and (d) ice calving expressed as sea-level-equivalent (SLE) per year. Solid lines indicate the ensemble mean and shading the ensemble range. Different colors represent the SSP5-8.5 with historical (black line; period 1850–2150 CE), and SSP5-8.5_MWOFF (cyan line), SSP5-8.5_HFOFF (red line), SSP5-8.5_CMOFF (pink line), SSP5-8.5_HFCMOFF (orange line) and SSP5-8.5_MWHFCMOFF (blue line) simulations during the period 2014–2150 CE.

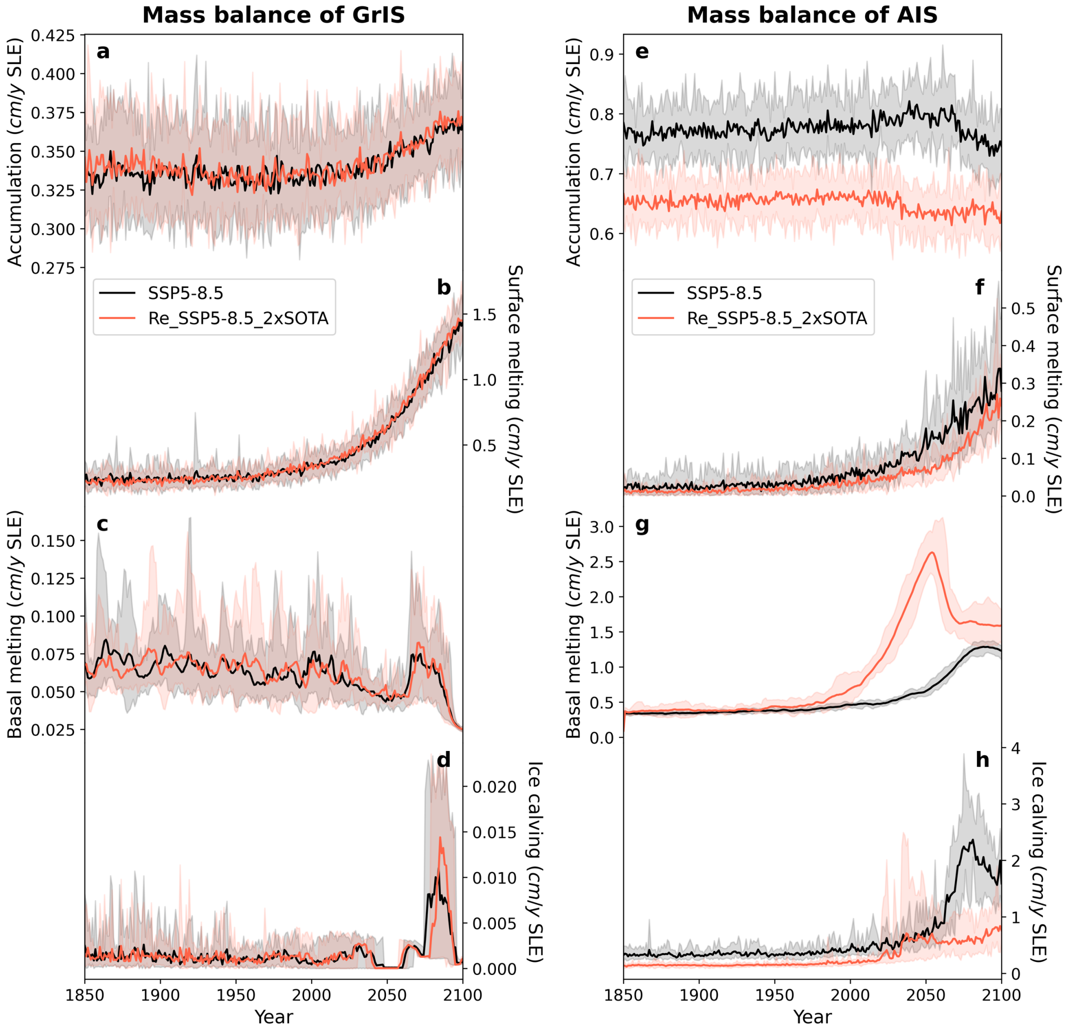

Supplementary figure S6 | Individual mass balance terms for Greenland ice-sheet (GrIS) and Antarctic ice-sheet (AIS) by subsurface Southern Ocean warming. (a-d) represent the individual GrIS mass balance terms for (a) the accumulation, (b) surface melting, (c) basal melting and (d) ice calving expressed as sea-level-equivalent (SLE) per year; (e-h), same as (a-d), but for AIS. Solid lines indicate the ensemble mean and shading the ensemble range. Different colors represent the SSP5-8.5 (black line) and Re_SSP5-8.5_2xSOTA (red line).

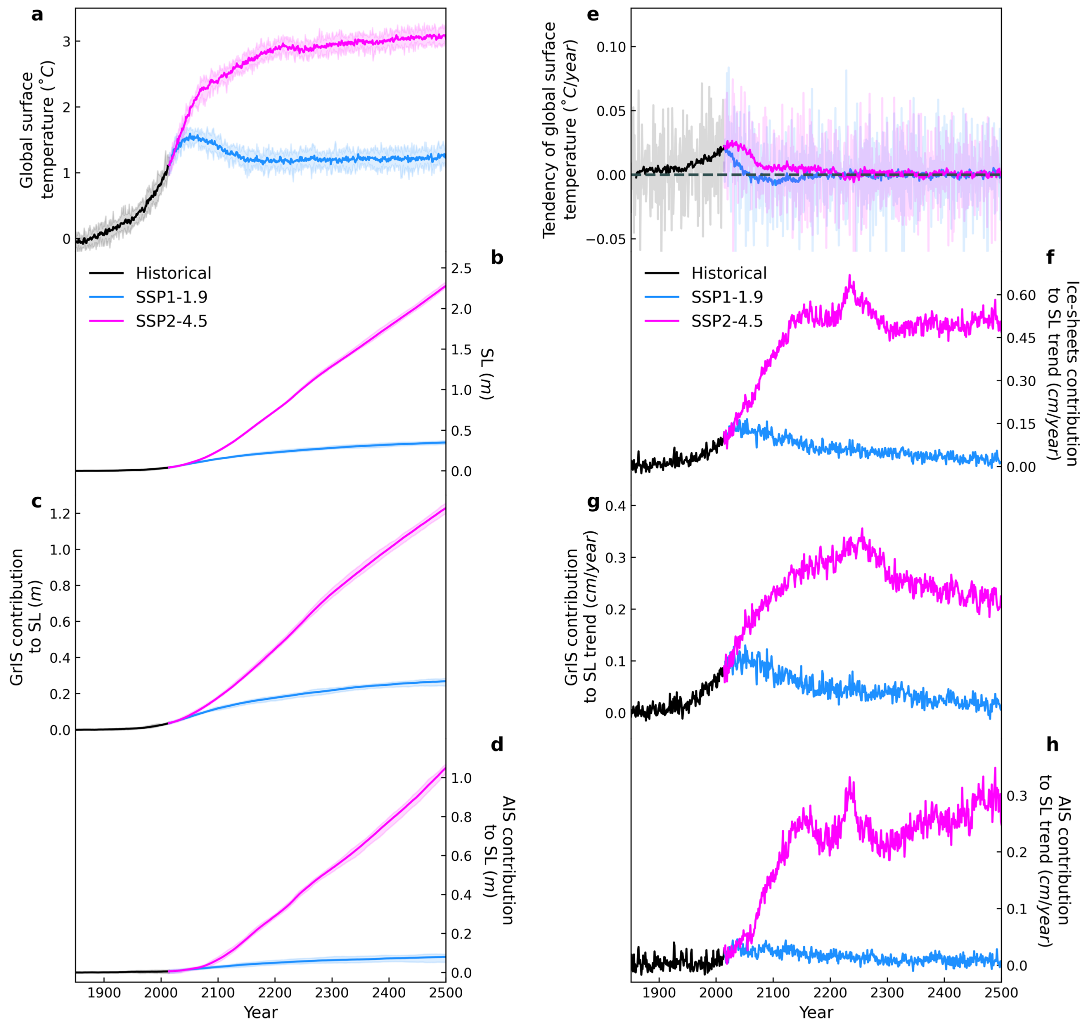

Supplementary figure S7 | Long-term Global surface temperature and sea-level (SL) projections, and their tendencies. (a-d) annual anomalies (relative to the 1850–1900 CE mean) of (a) the global surface temperature, (b) SL, and (c) SL contributions from the Greenland ice-sheet (GrIS) and (d) Antarctic ice-sheet (AIS). (e-h) are the respective time derivatives of (a-d) (change per year). Solid lines of (a-d) indicate the ensemble mean and shading the ensemble range. The solid line in (e) represents the 19-year moving average of the time derivative of global surface temperature, with the dashed line indicating 0 °C/year. Different colors represent the historical (black line; period 1850–2014 CE), and SSP1-1.9 (blue line) and SSP2-4.5 (pink line) simulations during the period 2014–2500 CE.

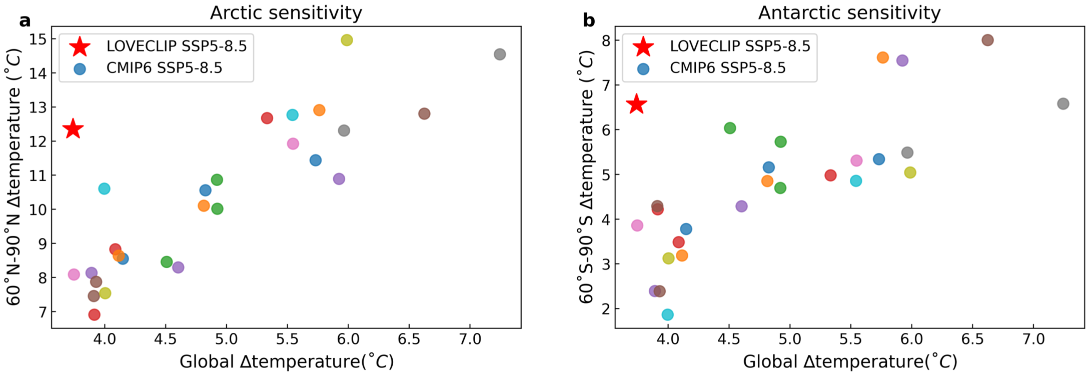

Supplementary figure S8 | Arctic and Antarctic sensitivities compared to global surface temperature increase by the end of 21st century. (a) Arctic amplification of LOVECLIP and CMIP6 under the historical and SSP5-8.5 scenarios. Δtemperature is the anomalous mean surface temperature in 2090-2100 relative to 1850-1900. (b) same as (a), but for Antarctic amplification. The Arctic region is defined as latitudes from 60ºN to 90ºN, and the Antarctic region from 60ºS to 90ºS.

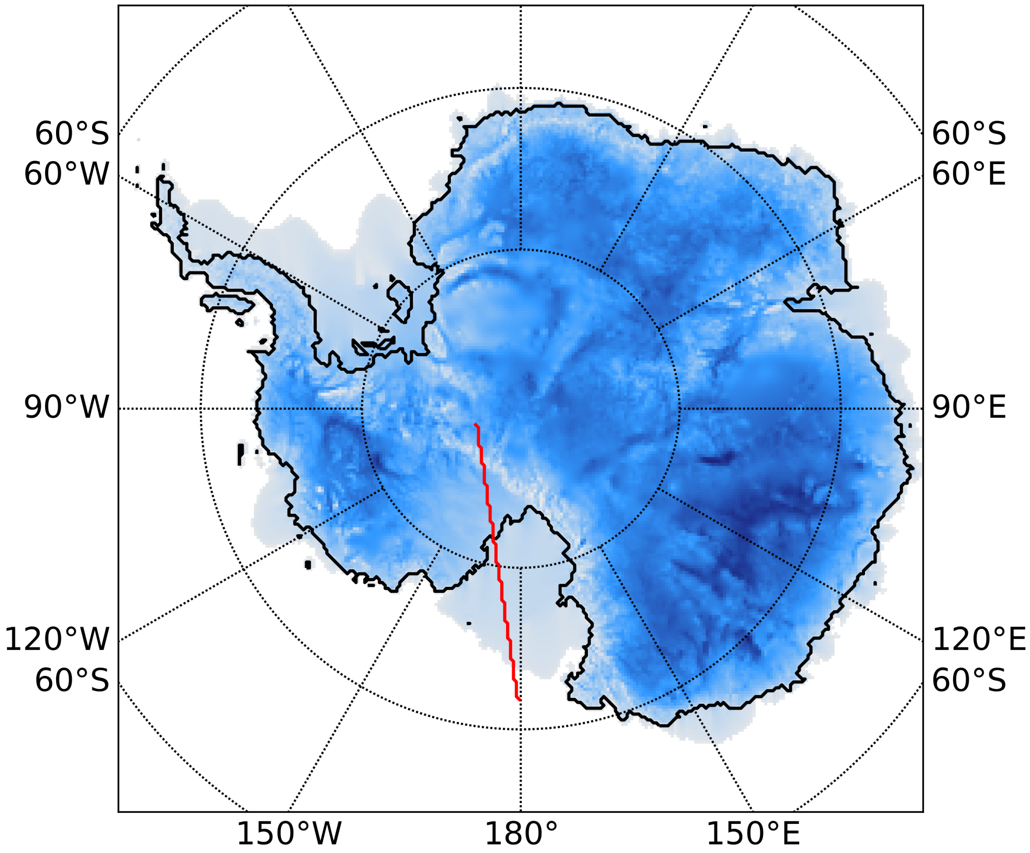

Supplementary figure S9 |Transection of Ross ice-shelf. The red line in this map indicates the location of Ross ice-shelf transection shown in figure 6.

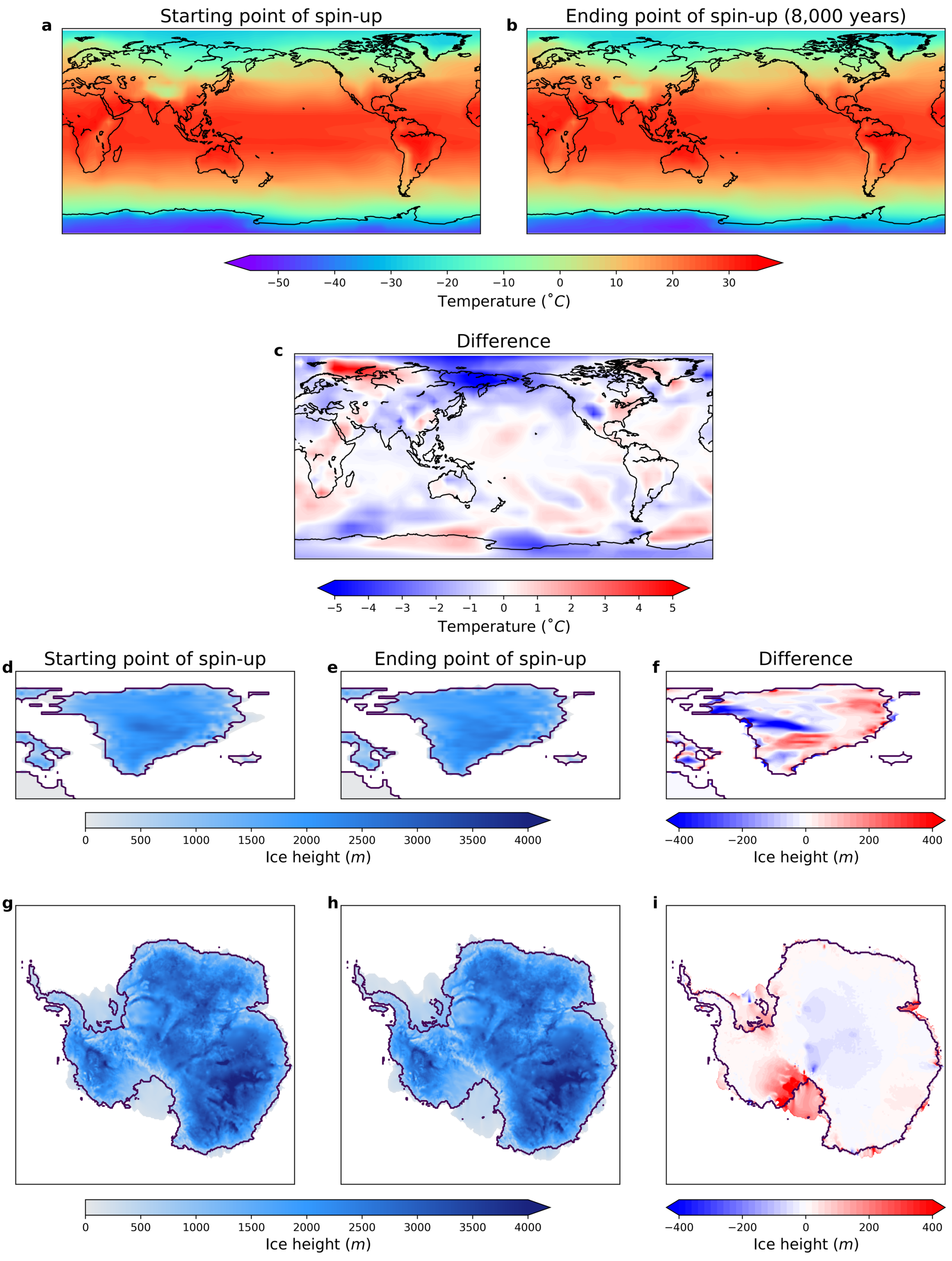

Supplementary figure S10 | Global temperature and ice height of Greenland ice-sheet (GrIS) and Antarctic ice-sheet (AIS) by the spin-up simulation. (a-c) global surface air temperature at (a) the starting point, (b) ending point and (c) difference between (b) and (a). (d-f) ice height of the GrIS at (d) the starting point, (e) ending point and (f) difference between (e) and (d); (g-i) same as (d-f), but for AIS.

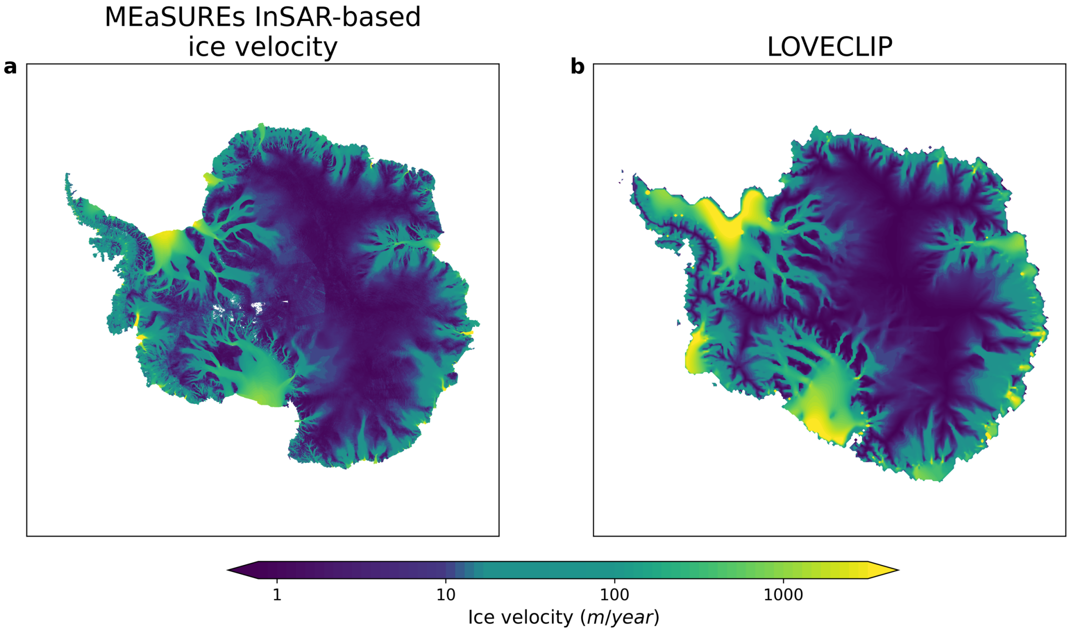

Supplementary figure S11 | Ice velocity over Antarctica. (a) Annual average of the 1996-2016 ice velocity over Antarctica (a) observed NSIDC-0484 satellite data[1,2] and simulated by (b) LOVECLIP.

Supplementary Table 1 | List of experiments conducted with LOVECLIP. This table shows the experiments that we conducted in this study. Ensembles of 10 members with different initial conditions were simulated for historical and SSP experiments, with the initial conditions taken from the last 100 chunks of the spin-up run.

Experiments	Set-up	Period (years)	
Spin-up	Asynchronous time coupling	LOVECLIM	PSUIM
		600+2000	6000+2000
CTR	Control simulation with pre-industrial CO_2 concentrations	5000	
Historical	Fully-coupled	165 (1850–2014 CE)	
SSP1-1.9	Fully-coupled	486 (2015–2500 CE)	
SSP2-4.5	Fully-coupled	486 (2015–2500 CE)	
SSP5-8.5	Fully-coupled	136 (2015–2150 CE)	
SSP5-8.5_MWOFF	Ignored AIS meltwater discharge	136 (2015–2150 CE)	
SSP5-8.5_HFOFF	Ignored AIS hydrofracturing process	136 (2015–2150 CE)	
SSP5-8.5_CMOFF	Ignored AIS ice-cliff failure process	136 (2015–2150 CE)	
SSP5-8.5_HFCMOFF	Ignored AIS hydrofracturing and ice-cliff failure process	136 (2015–2150 CE)	
SSP5-8.5_MWHFCMOFF	Ignored both AIS meltwater discharge, hydrofracturing and ice-cliff failure process	136 (2015–2150 CE)	
Re_SSP5-8.5_2xSOTA	Doubled 1850 year subsurface SO temperature anomaly	251 (1850-2100 CE)	
Re_SSP5-8.5_2xSOTA_MWOFF	Doubled 1850 year subsurface SO temperature anomaly and ignored AIS meltwater discharge	86 (2015-2100 CE)	

References

1 Mouginot, J., Rignot, E., Scheuchl, B. & Millan, R. Comprehensive annual ice sheet velocity mapping using Landsat-8, Sentinel-1, and RADARSAT-2 data. *Remote Sensing* **9**, 364 (2017).

2 Rignot, E., Mouginot, J. & Scheuchl, B. MEaSUREs InSAR-based Antarctica ice velocity map, version 2. *Boulder, Colorado USA. NASA National Snow and Ice Data Center Distributed Active Archive Center. doi: https://doi. org/10.5067/D7GK8F5J8M8R* (2017).
